# Infertility misperception and improper health-seeking behavior between urban and rural areas

**DOI:** 10.1371/journal.pone.0312456

**Published:** 2025-04-24

**Authors:** Berli Kusuma, Achmad Kemal Harzif, Mila Maidarti, Yudianto Budi Saroyo, Hariyono Winarto, Alfa Putri Meutia

**Affiliations:** 1 Department of Obstetrics and Gynecology, Faculty of Medicine Universitas Indonesia - dr. Cipto Mangunkusumo General Hospital, Jakarta, Indonesia; 2 Reproductive Immunoendocrinology Division, Department of Obstetrics and Gynecology, Faculty of Medicine Universitas Indonesia - dr. Cipto Mangunkusumo General Hospital, Jakarta, Indonesia; 3 Human Reproduction, Infertility, and Family Planning Cluster, Indonesia Reproductive Medicine Research and Training Center, Faculty of Medicine, Universitas Indonesia, Jakarta, Indonesia; UCSI University, Malaysia

## Abstract

**Objectives:**

The prevalence of infertility among reproductive-age couples in Indonesia is around 10-15%. Lack of understanding, misleading myths, and negative attitudes could result in improper behavior. This study aims to reveal the discrepancy between perception and behavior towards infertility in urban and rural areas in Indonesia.

**Materials and methods:**

A cross-sectional study using an internet-based questionnaire was given to 408 individuals, divided into two groups, Java and outside Java, representing urban and rural populations. The study included Indonesian citizens over 18 who were willing to participate, encompassing individuals of both genders, regardless of their fertility status. All participants completed the questionnaire from October 2020 to April 2021.

**Results:**

Half of the respondents from both groups consider infertility a disease. All respondents have excellent access to information. Although more than 80% of subjects from each group had been exposed to infertility information, a better understanding was observed in the urban community. Most subjects answered that smoking is the leading risk factor for infertility, followed by stress and advanced age. More respondents in rural areas have the wrong perception that stress causes infertility. Furthermore, they seek unwarranted advice, as 19.5% came to midwives and only 9.1% came to general practitioners. This study showed that 35.6% of subjects in the urban group and 41.6% in the rural group are considered late to seek healthcare assistance. Most respondents from both groups accept using Assisted Reproductive Technology and fertility-enhancing drugs as treatment options.

**Conclusion:**

Infertility misconceptions are more prevalent in rural groups than in urban groups. Fertility education among both groups needs to be improved to optimize the chance of conceiving and having a healthy baby.

## Introduction

Infertility is a health problem that receives less attention. Hardee et al., in their study, stated that infertility is one of the six neglected maternal morbidities in developing countries that impacts all aspects of a person’s life, creates negative stigma and unstable household life, and can cause anxiety and depression [1–3].

Research from 47 demographic and health surveys in developing countries in 2002 showed that 186 million women of reproductive age who have been married had infertility problems. This number accounts for more than a quarter of reproductive-aged women who have been married in the country [2,4]. The prevalence of infertility among reproductive-age couples in Indonesia is around 10–15% [5,6]. Unfortunately, the magnitude of infertility is not accompanied by a good perception of infertility. A study conducted in Indonesia found a limited understanding of infertility in Jakarta and Sumba. There is a lack of information, false beliefs, and a negative attitude about infertility. About 56.3 and 49.6% of participants in Jakarta and Sumba do not regard infertility to be a disease, and some do not believe that infertile couples require treatment. Some individuals believe that infertility is inherent and predetermined by God from the beginning [6]. Research in six European countries shows that only 38% of the population considers infertility a disease. This problem is getting more significant with the trend of marrying at an older age. Data from the Province of British Columbia, Canada, shows the proportion of mothers who are married at age > 30 years increased from < 7% in 1968 to 44% in 2005 [7]. This trend does not only occur in developed countries such as Canada, Europe, Australia, and the United States but also developing countries such as Indonesia [5,8–10].

Public perception can influence behavior to seek medical help for infertility. Lack of understanding, misleading myths, and negative attitudes could result in improper behavior. This study aims to reveal the discrepancy between perception and behavior towards infertility in urban and rural areas in Indonesia.

## Materials and methods

This research is a descriptive, cross-sectional study among conveniently sampled individuals using an internet-based questionnaire shared by communication platforms and social media. Data collected from the subjects were divided into two groups based on the residency of the subjects. Java, as the most populated center of economy and development, with advanced health care facilities, represents the urban area. While outside Java represents the rural areas of Indonesia.

The inclusion criteria for the study were Indonesian citizens over 18 who were willing to participate, encompassing individuals of both genders, regardless of their fertility status. The data was collected from an internet-based questionnaire from 1st October 2020 to 30th April 2021.

The questionnaire was generated based on a literature study [5–7,11–14]. The initial question set consists of 38 items. Since the questionnaire aims to capture various public perceptions regarding infertility, no statistical tests were conducted to determine the validity and reliability. The preparation of the research questionnaire is carried out in two phases. Phase I involves experts drafting and reviewing the questionnaire items to produce the primary questionnaire. A literature review is conducted to find questions used in other relevant studies to understand the subjects’ perceptions of the causes, access, and treatment of infertility. This is followed by phase II, which consists of collecting quantitative data and conducting validation tests on the questionnaire. In phase II of the questionnaire preparation, a pilot test of the questionnaire is conducted by distributing the primary questionnaire from Phase 1 to 30 respondents. Data is then collected to determine if respondents have difficulty understanding any of the questions. Questions that are difficult to understand will be revised for language and retested until a final questionnaire is produced.

Questionnaire validation was carried out in two stages. In the first stage, content validity was done based on expert panel discussions. From this panel discussion, options were added to the question items. Furthermore, a trial of the questionnaire was carried out on 42 subjects. Transcultural validity is carried out by asking for feedback from subjects to assess the similarity of content, namely whether each question item is relevant to the culture being studied and the similarity of concepts based on the same theoretical concepts in each culture. From this feedback, two more questions were added to the questionnaire.

Collected data were analyzed using IBM Statistical Package for Social Science (SPSS) version 25. We analyzed the sociodemographic characteristics of patients using descriptive statistics. Categorical data of sociodemographic factors and perception of infertility were analyzed using the Chi-square test. This study used a 5% error bound and 95% confidence interval limit.

This study was approved by the Research Ethics Committee of the Faculty of Medicine, Universitas Indonesia, with ethical clearance number KET-599/UN2.F1/ETIK/PPM.00.02/2020 and performed in accordance with the principles of the Declaration of Helsinki. Informed consent was obtained from all subjects before the study started.

## Results

In this study, 408 subjects filled out the questionnaires and were included. The study subjects were dominated by 296 females (72.5%), with 112 male subjects (27.5%). Most respondents are Muslim (68.4%) and have D4/S1 education (70.8%). A total of 156 (38.2%) subjects were married. Of the married subjects, 110 subjects already had children, while 46 subjects did not have children. Of all those who were married, 54 (34.6%) subjects had infertility after more than two years of marriage (Table 1).

**Table 1:** Subject characteristics.

Variables	(N)	(%)
Age (years old)		
<35 years old	379	92.9
≥35 years old	29	7.1
Gender		
Male	112	27.5
Female	296	72.5
Religion		
Islam	279	68.4
Christian	79	19.4
Catholic	35	8.6
Hindu	4	1
Buddhist	11	2.7
Education		
Senior high school	28	6.9
D3	63	15.4
D4/S1	289	70.8
S2	26	6.4
S3	2	0.5
Job		
Student	89	21.8
Government employee	50	6.1
Private employee	180	22.1
Army/Police	7	0.9
Housewife	15	1.8
Doctor	48	5.9
Nurse/Midwife/Pharmacist	19	2.3
Marital status		
Married	156	38.2
Unmarried	252	61.8
Number of children		
No child	46	29.5
1 child	71	45.5
2 children > 2 children	27 12	17.3 0.77
History of infertility		
Yes	54	34.6
No	102	65.4
Insurance		
No insurance	47	11.5
Government insurance	281	68.9
Other insurance	80	19.6

D3: Associate degree, D4: equivalent to bachelor’s degree, S1: bachelor’s degree, S2: master’s degree, S3: doctoral degree.

### Information access

Access to information from the 408 subjects who participated in the study is described in the following table (Table 2).

**Table 2:** Information access.

Variables	(N)	(%)
Read newspaper		
Yes	203	49.8
No	205	50.2
Watch television		
Yes	292	71.6
No	116	28.4
Listen to the radio		
Yes	175	42.9
No	233	57.1
Access to newspaper, TV, and radio		
Yes	395	96.8
No	13	3.2
Internet user		
Yes	408	100
No	0	0
Internet access in the last 12 months		
Yes	406	99.5
No	2	0.5
Internet access frequency		
<5 days/week	381	93.4
>5 days/week	27	6.6
Access to infertility information		
Yes	353	86.5
No	55	13.5
Source of infertility information		
Social Media	265	65
Website/internet	301	73.8
Newspaper/magazine	63	15.4
Family/college	110	27
No	31	7.6
		
Topics of Infertility Information		
Sperm analysis	158	38.7
Sexual transmitted disease	166	40.7
Smoking, alcohol, and drug use	291	71.3
Physical or psychological stress	244	59.8
Body weight	234	57.4
Age	213	52.2
Genetics	2	0.5
Electronics	1	0.2
Cat feathers	1	0.2
General topic	1	0.2
Never access infertility information	40	9.8

All subjects generally have good access to information from newspapers, television, radio, and the internet. The internet is the most widely used line of information, with 100% having internet access. Access to information through newspapers and radio has begun to be abandoned, with only 175 subjects listening to the radio and 203 reading newspapers. Access to information through television is still relatively high at 71.6%.

### Access to information by domicile (Java and outside Java)

Access to information is generally good in Java and outside Java. All respondents have access to sources of information from the internet, television, newspapers, or radio. The source of information accessed by most respondents is the internet (all respondents access information from the internet), followed by television (three-quarters of respondents). The proportion of respondents who access radio and print media is almost the same, one-third of all respondents. Radio is more desirable for respondents in the Java region than outside Java (47% versus 35%).

More than 80% (86.5%) of respondents said they had received information regarding infertility from various sources. Meanwhile, about a tenth of respondents (9.8%) had never received any information about infertility. The topic of infertility information that appeared the most was the effect of smoking, alcohol, and drugs on infertility (71.3%).

### Perception of the causes of infertility

While the understanding of infertility as a disease has not been entirely accepted, respondents who live in Java and outside Java show different understandings of secondary infertility. In both groups, the majority of respondents considered smoking to be a cause of infertility (Table 3).

**Table 3:** Association between domicile and perception of the causes of infertility.

	Domicile		P-value
	Java	Outside Java	
Infertility as a disease			0.400
Yes	124 (48.8%)	77 (50%)	
No	92 (36.2%)	61 (39.6%)	
Don’t know	38 (15%)	16 (10.4%)	
Possibility of becoming infertile after having a child			<0.001
Yes	179 (70.5%)	83 (53.9%)	
No	24 (9.4%)	46 (29.9%)	
Don’t know	51 (20.1%)	25 (16.2%)	
Main factor contributing to infertility			0.023
Smoking	87 (34.3%)	58 (37.7%)	
Contraception pill usage	15 (5.9%)	10 (6.5%)	
Psychological/physical stress	67 (26.4%)	57 (37%)	
Advance age	46 (18.1%)	16 (10.4%)	
Don’t know	39 (15.4%)	13 (8.4%)	
Age when fertility decrease			0.181
>30 years old	23 (9.1%)	15 (9.7%)	
>35 years old	109 (42.9%)	52 (33.8%)	
>40 years old	122 (48%)	87 (56.5%)	

### Perceptions of access to infertility treatment

For the respondents domiciled in Java, specialist doctors and general practitioners are the health workers most often referred to help overcome infertility problems (Table 4). In contrast, for the respondents outside Java, the dominant choices were specialist doctors and midwives.

**Table 4:** Association between domicile and perceptions of access to infertility treatment.

	Domicile		P-value
	Java	Outside Java	
First attempt for treatment			0.012
Specialist	181 (71.3%)	107 (69.5%)	
General practitioner	43 (16.9%)	14 (9.1%)	
Midwife	29 (11.4%)	30 (19.5%)	
Alternative medicine/shaman	1 (0.4%)	3 (1.9%)	
Next attempt for treatment			0.073
Specialist	233 (91.7%)	144 (93.5%)	
General practitioner	1 (0.4%)	0 (0%)	
Midwife	9 (3.5%)	0 (0%)	
Alternative medicine/shaman	11 (4.3%)	10 (6.5%)	

### Perceptions of infertility

Both groups revealed that both husbands and wives had to get treatment together to overcome infertility problems (Table 5). Acceptance of drugs for the treatment of infertility was equally well received in both groups, but acceptance of IVF treatment was more acceptable for the group domiciled in Java. Fewer respondents outside Java are aware of the importance of treating infertility in the first two years of marriage. The percentage of respondents who wait for >  5 years reaches 15.6%. Corresponding to some fertility issues, infertility was not the reason for husbands or wives to divorce, and adoption might be an option for those who did not have a child.

**Table 5:** Association between domicile and perceptions of infertility.

	Domicile		P-value
	Java	Outside Java	
Infertility needs to be treated			0.008
Yes	239 (94.1%)	153 (99.4%)	
No	15 (5.9%)	1 (0.6%)	
First to be treated			0.848
Husband	11 (4.3%)	9 (5.8%)	
Wife	15 (5.9%)	10 (6.5%)	
Both husband and wife	225 (88.6%)	134 (87%)	
Don’t know	3 (1.2%)	1 (0.6%)	
Infertility drugs treatment acceptance			0.923
Agree	175 (68.9%)	109 (70.8%)	
Don’t agree	7 (2.8%)	4 (2.6%)	
Neutral	72 (28.3%)	41 (26.6%)	
IVF treatment acceptance			0.033
Agree	201 (79.1%)	104 (67.5%)	
Don’t agree	5 (2%)	5 (3.2%)	
Neutral	48 (18.9%)	45 (29.2%)	
Waiting time before beginning treatment			0.020
1-2 year(s)	156 (61.4%)	90 (58.4%)	
2-5 years	80 (31.5%)	40 (26.0%)	
>5 years	18 (7.1%)	24 (15.6%)	
Infertility as men’s reason to divorce his wife			0.119
Yes	7 (2.8%)	9 (5.8%)	
No	247 (97.2%)	145 (94.2%)	
Infertility as women’s reason to divorce her husband			0.302
Yes	8 (3.1%)	8 (5.2%)	
No	246 (96.9%)	146 (94.8%)	
Infertility as men’s reason to polygamy			0.338
Yes	29 (11.4%)	13 (8.4%)	
No	225 (88.6%)	141 (91.6%)	
Infertility as women’s reason to polygamy			0.980
Yes	15 (5.9%)	9 (5.8%)	
No	239 (94.1%)	145 (94.2%)	
Infertility as reason for adoption			0.882
Yes	70 (27.6%)	45 (29.2%)	
No	10 (3.9%)	7 (4.5%)	
Neutral	174 (68.5%)	102 (66.2%)	
To be blamed for infertility			0.278
Women	6 (2.4%)	1 (0.6%)	
Both husband and wife	19 (7.5%)	8 (5.2%)	
No one	229 (90.2%)	145 (94.2%)	

## Discussion

This study evaluates the perspective of infertility among individuals residing in urban and rural regions of Indonesia. Infertility misperception refers to an incorrect view or understanding of infertility, which can affect how individuals approach infertility treatment. Misperceptions about infertility may result in improper health-seeking behavior, which is characterized as consulting non-medical practitioners, receiving incorrect treatment, or taking no action at all [15]. Our investigation showed that 54 (34.6%) subjects had infertility after more than two years of marriage. This figure is relatively high compared to the estimated rate of infertility in Indonesia, which is estimated at 10–15% [6]. The high number of respondents who have children more than two years after marriage can also be caused by the tendency to delay having children, as research in Japan shows [16].

Even though access to information is very good, and most respondents said they had been exposed to information about infertility, understanding is still lacking, with better understanding observed in the urban community. This result is in line with previous research, which showed low knowledge about infertility and ignorance regarding access and appropriate treatment [17]. Misinformation about infertility can negatively affect a person’s decision to seek treatment and increase their chances of achieving pregnancy. Educational background may influence knowledge of infertility. Knowledge about infertility varies significantly across educational levels. Individuals with higher levels of education have a greater understanding of infertility. Literacy also affects knowledge about infertility. Illiterate individuals exhibit a significantly lower knowledge score than those who are literate [18].

About 36.2-39.6% of all respondents did not consider infertility as a disease. These results align with previous studies conducted in Jakarta and Sumba, Indonesia [6]. This misconception that infertility is not a disease also occurs globally. Research in six European countries shows that only 38% of the population considers infertility a disease [7]. The normalization of infertility may result in couples refraining from obtaining treatment [6].

Most respondents already understand that smoking and alcohol are related to infertility, but most fail to answer that old age is the leading cause of decreased fertility in married couples. These results are in accordance with various studies regarding knowledge of age on infertility. Knowledge of the impact of aging on infertility in many parts of the world is poor, even in highly educated populations [19]. Some misunderstandings were also shown by respondents in this study. Some respondents still consider stress and the use of contraception as the leading causes of infertility. The view that stress, both physical and psychological, can reduce infertility indicates that respondents pay too much attention to this risk factor [20]. The knowledge of infertility risk factors, including advanced age, smoking, alcohol consumption, obesity, and other lifestyle risk factors, can encourage couples to avoid these factors, thereby enhancing their fertility potential [18].

Regarding access to infertility treatment, about 70% of respondents will try to seek treatment from a specialist. However, in Indonesia, specialist doctors may not be accessible to everyone because infertility itself is not a health problem covered by the National Health Insurance System. Respondents in rural areas have sought unwarranted advice, as 19.5% came to midwives and only 9.1% came to general practitioners. Respondents who live outside Java may prefer to go to midwives because of their wider distribution compared to general practitioners, considering that the ratio of midwives is higher than that of general practitioners outside Java. As explained in the flow of infertility treatment by HIFERI (Himpunan Endokrinologi Reproduksi dan Fertilitas Indonesia/ the Indonesian Fertility and Endocrinology Association), general practitioners have a role in the initial examination of infertility. General practitioners can do initial management of infertility cases without complicating factors in patients less than 30 years old. General practitioners can identify ovulation problems as well as tubal and peritoneal risk factors and then provide initial treatment, such as lifestyle changes. The role of midwives in infertility seems very small. Referring to their curriculum and the role of midwives themselves, infertility is not a health problem that this profession can handle [21].

From this study, we found that most respondents from both groups accept the use of Assisted Reproductive Technology and fertility-enhancing drugs as treatment options. This finding aligns with a study conducted in Ghana, where most respondents viewed in vitro fertilization (IVF) favorably. Acceptance of IVF services is highly associated with educational status [22]. In this study, acceptance for IVF was lower among respondents from rural areas compared to respondents who lived in Java. This could be due to less information and fewer IVF practices in rural areas compared to urban areas.

Only 60% of all respondents will take treatment within 1–2 years; the rest delay it for up to or more than five years. This could be attributed to a lack of knowledge about infertility and the potential results of treatment. Other variables that may impact couples’ decision to pursue infertility treatment are accessibility and costs of treatment, negative stigma associated with infertility, and lack of spousal support [23]. The older age and the later treatment initiation are a problem because of the reduced possibility of pregnancy at the age of 35 and over. This problem is getting more significant with the trend of marrying at an older age. Data from the Province of British Columbia, Canada, shows that the proportion of married mothers aged > 30 years old increased from < 7% in 1968 to 44% in 2005. Data from Japan shows that the average age of women when having their first child increased from 2005, with an average of 29 to 30.7 years old in 2019 [7]. This trend occurs not only in developed countries such as Europe, Australia, and the United States but also in developing countries such as Indonesia [5,8–10].

This study showed that 38.6% of subjects in the urban group and 41.6% in the rural group are considered late to seek healthcare assistance, which is seeking treatment after waiting more than 2 years. This finding is similar to previous studies where about 40% of married couples are late in seeking infertility treatment. The percentage of couples who are late for treatment is found to be higher in respondents outside Java. For residents who live outside Java, the prognosis might be worse since by the time they decide to seek healthcare treatment, no proper facilities are available in their area. This may lead to delayed diagnosis and delayed treatment initiation for those in rural regions of Indonesia.

Infertility is not the reason accepted by the majority of respondents to divorce their spouse. Despite the equal contribution of male and female factors to infertility cases, women seem to be more affected socially by infertility. Women are commonly blamed for infertility, which causes emotional pain, societal humiliation, and rejection. Studies in the UK, USA, and Ghana found a high incidence of stigma affecting women experiencing infertility [24]. This may be due to the lack of knowledge regarding the medical causes and infertility treatment options observed among developing countries [6].

Adoption is considered one of the possible options for couples with infertility and may offer a better solution than divorce. Only 4% of respondents refused adoption for infertile couples. Most Indonesians think that married couples should have children. However, adoption is not a common practice. Data in Indonesia is still scanty, but in Karachi, Pakistan, only 6% of 400 infertile couples adopt children. There appears to be a feasible option for infertile couples to adopt children, but from some psychological perspective, adopting a child still cannot replace the feeling of having one’s child [6].

Various studies have shown that the lack of knowledge and awareness of factors causing infertility, such as age, contributes to the delay of couples having children [17]. In this study, access to information was excellent, with more than 80% of respondents exposed to infertility-related information. However, correct knowledge about infertility is relatively low. This discrepancy can be caused by untrustworthy sources of information about infertility, mainly obtained from internet websites. The accuracy of such information was not guaranteed. About 86.5% of respondents have read/heard about infertility, of which 74% are obtained from the internet, but knowledge about infertility is still low. Topics accessed by the majority of respondents were related to the effects of smoking, drugs, and stress on infertility. Few topics discussed the correlation between increasing age and decreasing fertility.

Compared to other countries, Indonesia does not have an infertility education program. Australia, Denmark, the United Kingdom, Belgium, Greece, Portugal, the Czech Republic, and Sweden are countries that have started fertility education programs. The Australian government funds educational programs in the country, including a website with 5 million visits annually and fertility learning modules for health care providers [25]. Denmark has set up a fertility counseling and assessment clinic [26,27]. The United Kingdom started fertility education in 2016 and is incorporating it into the curriculum in 2019. Other countries also already have fertility education websites [17].

This study is the first investigation that describes the public’s perceptions of infertility in Indonesia. The strength of the study is that it overcame geographical barriers and reached subjects in faraway regions by employing internet-based questionnaires. However, our study does have limitations. The study’s limitation is that it is likely not accessible to individuals from low-income backgrounds, as it is a survey that utilizes an internet-based questionnaire. Another limitation is the imbalance between male and female participants and the imbalance of the age groups.

## Conclusion

Misperceptions about infertility are more prevalent in rural groups compared to urban groups. Fertility education among both groups should be developed to achieve better fertility outcomes. Internet technology offers an effective method for education purposes.

## Supporting information

S1 File The supplementary material contains the detailed preparation process of the questionnaire used in the study. It describes the two phases of the questionnaire development, including the drafting and validation processes, as well as the specific questions used to measure public perception on infertility in both urban and rural areas. This document also outlines the sources and references used in developing the research.
(DOCX)
