# Supplementary material for: Infertility misperception and improper health-seeking behavior between urban and rural areas
Source: PLoS One. 2025 Apr 24;20(4):e0312456. doi: 10.1371/journal.pone.0312456 (PMC12021135; doi:10.1371/journal.pone.0312456)
Supplement: S1 File — The supplementary material contains the detailed preparation process of the questionnaire used in the study. It describes the two phases of the questionnaire development, including the drafting and validation processes, as well as the specific questions used to measure public perception on infertility in both urban and rural areas. This document also outlines the sources and references used in developing the research. (DOCX) [file pone.0312456.s001.docx]

**Supplementary Material:**

**Preparation of the questionnaire**

**Preparation of Research Instruments**

The instrument used to measure public perception in this study is a questionnaire. The preparation of the research questionnaire is carried out in two phases. Phase I involves experts drafting and reviewing the questionnaire items to produce the primary questionnaire. This is followed by Phase II, which involves collecting quantitative data and conducting validation tests on the questionnaire.

**Phase I**

In Phase I, the questionnaire items are drafted. A literature review is conducted to find questions used in other relevant studies to understand the subjects' perceptions of the causes, access, and treatment of infertility. In their study, to understand the differences in perceptions of infertility between urban and rural areas, Harzif et al. used the following questions:^1^

1. What causes infertility?

a) Menstrual disorders

b) Reproductive tract issues

c) History of urinary tract infections in women

d) History of urinary tract infections in men

e) Smoking

f) Use of birth control pills by women

g) Use of IUDs by women

h) Other supernatural/mystical reasons

i) Black magic/being cursed

j) Regular exercise

k) Psychological stress

l) Obesity

m) Genetic factors

n) Alcohol

2. Is infertility a disease?

3. Should infertility be treated?

4. Who should be examined first?

a) Husband

b) Wife

c) Both

5. If a couple already has biological children, is it possible for them to become infertile?

6. Where would you seek treatment if you had difficulty conceiving?

a) Doctor

b) Alternative medicine

c) Traditional healer

7. If treatment is unsuccessful, where would you seek further treatment?

a) Doctor

b) Alternative medicine

c) Traditional healer

8. If a woman cannot have children, do you think this is a reason for divorcing her?

9. If a woman cannot have children, do you think this is a reason for remarrying?

10. If a man cannot have children, is this a reason for divorcing his wife?

11. If a man cannot have children, do you think this is a reason for remarrying?

12. If a couple does not have children, should they consider adopting?

13. Who can be blamed for infertility issues in society?

a) Men

b) Women

c) Both

d) No one

14. Do you agree with in vitro fertilization (IVF)?

15. Are infertility treatments acceptable to the public?

Adashi et al., in their research, used questions regarding whether infertility is considered a disease, the definition of infertility, views on IVF programs, and views on the financing of IVF.^2^ Perceptions regarding the relationship between age and fertility were asked, as in the study by Bennett et al.^3^, which included questions about when a couple should seek medical help and when fertility begins to decline, as in the research by Bretherick et al.^4^

Questions from the Indonesian Basic Health Survey are used as a reference to obtain data on information access through the questionnaire, as follows:

Table 3.0.1 Indonesian Basic Health Survey Questionnaire

1. Do you read newspapers/magazines at least once a week?

a) Yes b) No

2. Do you watch TV at least once a week?

a) Yes b) No

3. Do you listen to the radio at least once a week?

a) Yes b) No

4. How is your access to information media?

a) Access to all three media at least once a week

b) No access to any media at least once a week

5. Have you ever used the internet?

a) Yes b) No

6. Have you used the internet in the last 12 months?

a) Yes b) No

7. How frequently do you use the internet in the last month?

a) Almost every day

b) At least once a week

c) At least once a month

d) Not at all

**Phase 2**

In Phase 2 of the questionnaire preparation, a pilot test of the questionnaire is conducted by distributing the primary questionnaire from Phase 1 to 30 respondents. Data is then collected to determine if respondents have difficulty understanding any of the questions in the questionnaire. Questions that are difficult to understand will be revised for language and retested until a final questionnaire is produced.

**References**

1. Harzif AK, Santawi VPA, Wijaya S. Discrepancy in perception of infertility and attitude towards treatment options: Indonesian urban and rural area. Reprod Health. 2019;16(1):126.
2. Adashi EY, Cohen J, Hamberger L, Jones HW, Jr., de Kretser DM, Lunenfeld B, et al. Public perception on infertility and its treatment: an international survey. The Bertarelli Foundation Scientific Board. Hum Reprod. 2000;15(2):330-4.
3. Bennett LR, Wiweko B, Hinting A, Adnyana IP, Pangestu M. Indonesian infertility patients’ health seeking behaviour and patterns of access to biomedical infertility care: an interviewer administered survey conducted in three clinics. Reproductive Health. 2012;9(1):24.
4. Bretherick KL, Fairbrother N, Avila L, Harbord SH, Robinson WP. Fertility and aging: do reproductive-aged Canadian women know what they need to know? Fertility and sterility. 2010;93(7):2162-8.
